# Chemical Components, Emission Dynamics, and External Immune Functions of Red Palm Weevil Larval Volatiles in Response to Changes in Developmental Stages and Pathogen Stress

**DOI:** 10.3390/insects16121266

**Published:** 2025-12-13

**Authors:** Can-Hui Ding, Wen-Qing You, Zong-Wei Zheng, Yu-Chen Pu, Li-Na Xu, You-Ming Hou, Yue Zhang, Cong Ou-Yang

**Affiliations:** 1 State Key Laboratory of Agricultural and Forestry Biosecurity, Fujian Agriculture and Forestry University, Fuzhou 350002, China; 12302003023@fafu.edu.cn (C.-H.D.); youwenqing02@163.com (W.-Q.Y.); 17750357920@163.com (Z.-W.Z.); kunka-lina@outlook.com (L.-N.X.); 2 Key Laboratory of Landscape Plants with Fujian and Taiwan Characteristics of Fujian Colleges and Universities, School of Biological Science and Biotechnology, Minnan Normal University, Zhangzhou 363000, China; 15138303981@163.com (Y.Z.); 15083862415@163.com (C.O.-Y.); 3 Fujian Provincial Key Laboratory of Insect Ecology, College of Plant Protection, Fujian Agriculture and Forestry University, Fuzhou 350002, China

**Keywords:** antimicrobial efficacy, external immune defense, pathogen infection, *Rhynchophorus ferrugineus*, SPME-GC-MS, volatile chemicals

## Abstract

Chemical defenses help insects resist pathogen infection. The volatile compositions, emission patterns, and external immune functions of the red palm weevil (RPW), a major invasive pest now established in numerous countries including the Mediterranean Basin, North Africa, Middle East, and parts of Latin America and the Caribbean, are largely unknown. In this study, we examined RPW larval volatiles, analyzing their emission patterns across developmental stages and under pathogen stress from feeding *Metarhizium anisopliae*. RPW larvae shared a number of volatile components across stages, but the emission dynamics were significantly different. These volatile chemicals were primarily alcohols, phenols and aromatic hydrocarbons, and styrene was the predominant volatile. Furthermore, pathogen stress induced distinct volatile profiles: phenylacetaldehyde unidirectionally decreased, whereas *n*-nonanol, 4-ethylguaiacol, 2-phenylethanol, hexanal, and benzophenone levels increased. Antimicrobial analysis showed that these upregulated compounds broadly inhibited fungal and bacterial growth. Therefore, our results illustrated the immune defense role of RPW larval volatiles and their potential bioactive compounds, including *n*-nonanol, 4-ethylguaiacol, 2-phenylethanol, hexanal, and benzophenone.

## Simple Summary

The red palm weevil is a highly devastating invasive beetle that kills palm trees, causing significant economic losses and ecological destruction. Controlling this pest with chemical pesticides is difficult, and even natural pathogens like fungi are often ineffective because the insect has a strong immune defense. Our research discovered that the weevil’s larvae release a mix of volatile chemicals into the surrounding environment. We found that the amount and type of these chemicals change as the larvae grow and, importantly, when they are attacked by a harmful fungus. Several of these increased chemicals, including compounds like 2-phenylethanol and hexanal, were shown to powerfully inhibit the growth of fungi and bacteria in our lab tests. This reveals a previously unknown “external immune system”, where the weevil essentially uses airborne chemicals to disinfect its immediate surroundings and fight off infections. Understanding this natural defense provides new ideas for developing more effective and environmentally friendly methods to control this devastating pest by potentially disrupting its protective chemical shield.

## 1. Introduction

The red palm weevil (RPW), *Rhynchophorus ferrugineus* (Coleoptera: Curculionidae), is a destructive pest that has severely invaded palm planting areas in southern China in recent years [1,2]. This species is reported to have originated in South Asia and Melanesia and was first discovered in India in 1891 [3]. Since the 1980s, the RPW has invaded and spread on a large scale to the Middle East, Southeast Asia, North Africa, southern Europe, and other areas, mainly through seedling transport [4]. The RPWs are holometabolous insect with a life cycle comprising egg, larva, pupa, and adult stages and the larval stage serves as the most damaging stage. Adult RPWs lay eggs in the scars and cracks on the plant surface, while the larvae bore into the trunk to form tunnels, resulting in tissue necrosis and rot, poor tree growth, and in severe cases, stem hollowness and the death of many palms, thus causing significant economic losses to the palm industry and landscape development of the invaded area [5,6,7].

The parts damaged by RPW are concealed, and traditional chemical pesticides and physical control are ineffective; therefore, RPW exhibits strong adaptability to the environment [1,5,8,9]. On the contrary, biological control is particularly important for the sustainable control of RPW [10]. Extensive biological control studies on *Heterorhabditis indica* [11], *Steinernema carpocapsae* [12], *Bacillus thuringiensis* [13], *Beauveria bassiana* [14,15], and *Metarhizium anisopliae* [16,17] have been conducted, but none of these pathogen resources have been applied to large-scale control practices. Moreover, the control efficacy of these pathogens on RPW is largely different and is affected by various factors, including the type of pathogen, the concentration of the pathogen, the period of infection, the developmental stage of the host, and early infection experience. The mechanism involved is mainly the immune defense of the host. When a pathogen infects a host, the host must initiate a series of immune response processes to evade the potential threat [18,19], which severely weakens the control efficacy of pathogens against pests.

Insects have evolved diverse immune strategies, among which external immunity plays a critical role. This defense mechanism involves the active secretion of chemical substances into the external environment to suppress or eliminate pathogenic threats [20,21]. Specifically, these externally released compounds can regulate the microbial composition in the insect’s immediate surroundings, thereby serving as a frontline chemical barrier [22,23,24].

Most immune defensive compounds do not have target specificity and have broad-spectrum efficacy against a variety of parasites [25]. Most of these compounds are volatiles released by defensive organs, such as acids, acetaldehyde, aromatic ketones, and terpenes with low molecular weights [26]. These volatiles have certain immune functions against pathogens and can exert antimicrobial effects not only internally but also externally after being released through various insect glands [27,28]. The secretions of the larvae of the genus *Chrysomela* have antimicrobial activity against entomopathogenic bacteria and fungi [29]. The substances on the epidermal surface of RPW eggs and larvae have an inhibitory effect on the growth of Gram-positive bacteria [30]. In addition, some compounds in insect volatiles also function as pheromones [31,32]. In most cases, the amount of pheromones released by insects is relatively small, and immune defense requires the release of high concentrations of compounds, which limits the functional overlap between immunity and pheromones to a certain extent.

Most recent studies on volatiles and their antimicrobial efficacy have focused on the plant defensive response to pests or the plants themselves. However, previous studies have confirmed that insect external volatiles contain active antimicrobial components. The larvae of the brassy willow leaf beetle *Phratora vitellinae* release volatile substances from their glands to fight against pathogens in the living environment and prevent the germination of fungal conidia on their cuticles [33]. Gross et al. [33] reported that salicylaldehyde, the main active component of the volatiles of *P. vitellinae*, was released from the opening of the larval glands and can inhibit the growth of *B. thuringiensis*. If the contents of the dorsal glands are removed, the concentration of volatiles around the larvae temporarily decreases, and the susceptibility of the larvae infected with *B. bassiana* and *M. anisopliae* increases, which can lead to increased mortality when exposed to fungal pathogens [33]. Gross et al. [34] detected phenol, α-pinene, and benzaldehyde in the headspace volatiles of ladybird beetle *Harmonia axyridis*, all of which inhibited the growth of *Escherichia coli*, *Micrococcus luteus*, and *Saccharomyces*. Similarly, volatiles from migratory locust (*Locusta migratoria*) and grasshopper (*Oedaleus asiaticus*), containing components like guaiacol, phenol, octanal, and decanal, have also demonstrated antifungal and antibacterial properties [31,35,36,37,38]. These findings further indicate that insect volatiles are expected to exert specific external immune functions when they are exposed to the microbial environment.

Factors such as sex, developmental stage, population density, and reproductive status of insects affect the emission patterns of individual volatiles [39,40]. On the one hand, the composition and absolute amount of locust volatiles change dynamically with sex, gregariousness, and widowhood [31]. On the other hand, old nymphs release fewer external volatiles than young nymphs do, whereas adults release significantly more external volatiles than do nymphs [41]. Moreover, as the weight of the insect increases, the amount of volatiles released also changes.

Although there is growing evidence that insect volatiles have antimicrobial activities, their role and importance in external immune defense are still not well understood. In this study, the chemical composition and emission dynamics of volatiles from RPW larvae in response to changes in developmental stage and external stress by pathogens were investigated via solid-phase microextraction–gas chromatography–mass spectrometry (SPME-GC-MS). We further elucidated the external immune function and potential biologically active compounds of the volatiles, thereby explaining the external defense of RPW larvae against pathogens. Therefore, the purpose of this study is to enhance the field control effect of pathogenic microorganisms from the perspective of destroying the immune system and to provide guidance for the development of new technologies for pest control based on immune regulation.

## 2. Materials and Methods

### 2.1. Insect Collection and Rearing

The colony of *R. ferrugineus* used in this study originated from adults trapped in 2019 on the campus of Fujian Agriculture and Forestry University (26°04’48” N, 119°14’24” E), Fuzhou City, Fujian Province, China. Mature males and females were kept in pairs in 330 mL clean plastic bottles (70 mm in diameter, 107 mm in height) containing sugarcane stem tissues (approximately 50 mm in length, 25 mm in width, 10 mm in height). Eggs were daily collected on moist degreasing cotton or filter papers. The newly hatched larvae were reared individually on a diet of fresh sugarcane stems every 7 d in Petri dishes (90 mm in diameter) until pupation and eclosion. In the laboratory, RPW adults were maintained at 25 ± 1 °C and 75 ± 5% relative humidity (RH) with a 12 h light/12 h dark schedule in a growth chamber, and a 24 h dark photoperiod was used for the other stages of the life cycle. After one generation of reproduction, offspring larvae at different developmental stages were selected, which referred to Pu et al. [21], including the fifth-instar (average 1.0 g), the seventh-instar (average 2.0 g), the ninth-instar (average 3.0 g), and the eleventh-instar (average 4.0 g), for further experiments.

### 2.2. Microbe Preparation for Infection and Inhibition Assays

The fungus *M. anisopliae* (Accession No. MF467274), a widespread entomopathogen, was previously isolated by Pu et al. [17] from RPW adults that died of natural infectious disease; this fungus can decrease larval fitness and cause significant larval mortality [17]. This strain was first grown on potato dextrose agar (PDA) medium (Shanghai Bioway Technology Co., Ltd., Shanghai, China) and incubated at 25 °C for 7~10 d until a large number of dark green conidia were harvested. Viable germinating conidia were then resuspended in sterile distilled water containing 0.01% Tween 80 to prepare fungal spore suspensions. Spore density was estimated using a hemocytometer and adjusted to required concentration of 1.0 × 10^5^ conidia/mL or 1.0 × 10^7^ conidia/mL according to the methods described by Sun et al. [42].

The bacterial strains *E. coli* CMCC44102 (Gram-negative) and *Staphylococcus aureus* CMCC(B)26003 (Gram-positive), provided by Shanghai Bioresource Collection Center, were selected as representative model organisms for the antibacterial assays and cultured overnight on Luria–Bertani (LB) broth medium (Beijing Solarbio Science & Technology Co., Ltd., Beijing, China) at 37 °C in a shaker at 200 rpm. To determine cell density, the number of bacterial colony forming units (CFUs) was counted by visualizing the LB agar (Beijing Solarbio Science & Technology Co., Ltd., Beijing, China) plates following the steps of Habineza et al. [43]. Finally, the bacterial suspension of 1.0 × 10^4^ CFU/mL was obtained.

### 2.3. Collection of RPW Larval Volatiles at Different Developmental Stages

**Figure 1:**
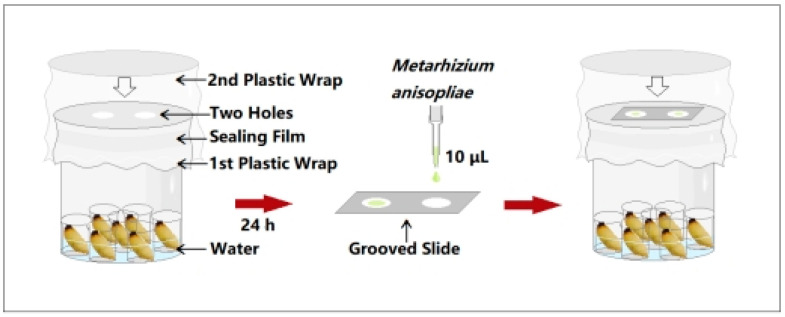
Collection and fungistatic experimental device for the release of external volatiles by red palm weevil (RPW) larvae.

Fifth-, seventh-, ninth-, and eleventh-instar RPW larvae of similar weights and sizes were selected as the experimental insects. To reduce the influence of food residues in the gut and mouth on the experimental results, each larva was placed separately in a clean box for 1 h to excrete the sugarcane residue from the gut and mouth. The larvae were subsequently rinsed five times with sterile water. Then, they were wiped with 75% alcohol on an ultraclean workbench and placed in a sterile disposable Petri dish to dry. To prevent the larvae from biting each other and affecting the test, seven small 10 mL beakers were placed in a large 500 mL beaker and then seven larvae at the same developmental stage were placed in the small beakers separately. A group without larvae was used as a blank control. The 500 mL beaker was sealed with plastic wrap, and then Parafilm was wrapped around the beaker to fix the plastic wrap in place. Next, scissors were used to cut out two circular holes with a diameter of 2 cm on the plastic wrap (the distance between the two circular holes refers to the distance between the two grooves of the double concave slide so that the grooves are aligned with the circular holes and completely contact the headspace environment of the collection beaker through the circular hole), and then the beaker was sealed again with plastic wrap to prevent the collected volatiles from escaping through the circular holes (Figure 1). The collection device was placed in a growth chamber to collect insect external volatiles for 24 h under normal larval growth conditions.

### 2.4. Assays on the Inhibitory Ability of Volatiles Against Pathogens

After 24 h of insect volatile collection, 10 μL of a suspension of *M. anisopliae* spores at a concentration of 1.0 × 10^7^ conidia/mL was applied to the grooves of a double concave slide. The upper plastic wrap of the volatile collection device was opened. The double concave slide was inverted and placed on the collection device after the grooves were aligned with the two holes of the lower plastic wrap, and then the collection device was immediately covered and sealed with the upper plastic wrap, so that the fungal solution was fully exposed to the environment of the volatiles released by the larvae (Figure 1). The collection devices were returned to the growth chamber under the same conditions for 12 h. The germination status of the *M. anisopliae* conidia was observed under a light microscope. Spores were considered to have germinated when the length of the germ tube was greater than half of the spore radius. A total of 5~10 different fields of view were selected from the two grooves to determine the spore germination rate. Each treatment was repeated five times. The external immune defense efficacy of RPW larval volatiles was measured by analyzing the differences in spore germination rates between the treatment groups (different larval developmental stages) and the control group.

### 2.5. Qualitative and Quantitative Analysis of Volatile Components

**Figure 2:**
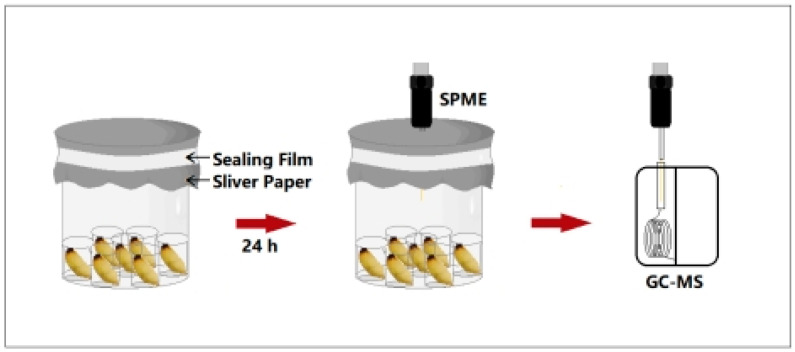
Experimental device for the determination of chemical components from RPW larval volatiles via SPME-GC-MS.

According to the method described in Wei et al. [31], the SPME-GC-MS (7980A-5975C, Agilent, Santa Clara, CA, USA) technique was used to determine the composition and content of external volatiles released by RPW larvae of different developmental stages. Three biological replicates were performed. Before the samples were measured, the heating program set by the GC-MS was started, and sampling with an empty needle was performed. First, the SPME tip was inserted into the GC injection port, and the fiber tip was pushed out. After aging the extraction tip for 15 min, the fiber tip was retracted and removed from the injection port. The sample bottle was placed under the fixture, the aged extraction tip was inserted into the sample vial, the handle of the extraction needle was fixed with a fixing clip, and the fiber tip was gently pushed out and then adsorbed for 1 h. During the 35 min procedure with an empty needle, the instrument was set for a new round of tests. After adsorption was complete, the fiber tip was retracted to the extraction tip. Using the same method, the sample was injected into the GC injection port, and the program was started for a new round of volatile measurement (Figure 2).

The GC conditions were as follows: the chromatographic column was an HP-5MS quartz capillary column (30 m × 0.25 mm × 0.25 μm), the carrier gas was high-purity helium, the flow rate was 1 mL/min, and the split GC injection mode was used. The injection port temperature was 250 °C. The initial column oven temperature was 50 °C and was maintained for 5 min, and the temperature was then increased to 250 °C at 10 °C/min and maintained for 10 min. The total program time was 35 min.

The MS conditions were as follows: interface temperature, 250 °C; EI ion source temperature, 200 °C; electron energy, 70 eV; detector voltage, 300 V; and mass scan range, 35–335 amu.

The mass spectra obtained from the external volatiles of the RPW larvae were compared with a standard mass spectrum library (FFNSC 1.2) to qualitatively determine the chemical composition. The mass spectral responses of the volatiles were measured via GC-MS. The raw data were then integrated and quantitatively analyzed using the GC/MS Solution workstation. The relative amount of each component is expressed as a percentage of the mass spectral ion peak area.

### 2.6. Detection of Volatiles Following Challenge of Larvae with External Pathogens

To achieve pathogen challenge, sugarcane slices evenly coated with 1 mL of a sublethal dose of 1.0 × 10^5^ conidia/mL of *M. anisopliae* spore suspension were fed to ninth instar RPW larvae. Tween 80 (0.05%) was used as a control. As the method mentioned above, after 24 h of treatment with or without pathogen stress, the external volatiles released by the individual were collected, and the chemical components were detected, thereby preliminarily screening potential external immune-active components of the RPW larvae involved in the response to pathogen stress. Four biological replicates were performed.

### 2.7. Screening of Immunocompetent Compounds in Volatiles

Five potential active chemicals from the volatiles released by the RPW larvae were selected for external immune function, including *n*-nonanol, 4-ethylguaiacol, 2-phenylethanol, hexanal, and benzophenone. The spore germination method and the Oxford cup method were used to verify the inhibitory effects of a single component on fungi such as *M. anisopliae* and bacteria such as *E. coli* and *S. aureus*, respectively.

In the fungal growth inhibition experiments, the pure chemical product was dissolved in absolute ethanol and diluted twofold to produce six solutions with different concentrations, including 1 mg/mL, 0.5 mg/mL, 0.25 mg/mL, 0.125 mg/mL, 0.0625 mg/mL, and 0.03125 mg/mL, in a 1.5 mL centrifuge tube. First, 10 μL of each solution was added dropwise into each groove of a double concave slide, and 10 μL of absolute ethanol was used as a control. Next, 10 μL of *M. anisopliae* spore suspension at 1.0 × 10^7^ conidia/mL was added and mixed well. The double concave slides were subsequently placed in a wet box with sterile water at the bottom, and the box was placed in a biochemical incubator at 25 °C for 12 h. The germination status of the *M. anisopliae* conidia was observed under a light microscope, and the spore germination rate was calculated. This experiment was repeated six times. The minimum inhibitory concentration (MIC) of each compound was further evaluated by comparing the inhibitory effects of the chemicals at different concentrations on the fungi.

In the bacterial growth inhibition experiments, the pure chemical product was dissolved in absolute ethanol to prepare a solution at a concentration of 0.1 mg/mL in a 1.5 mL centrifuge tube. An improved version of the inhibition zone method described in Pu et al. [44] was used in this study. First, 15 mL of LB agar medium was poured into a disposable sterile Petri dish. When the medium had cooled to approximately 45 °C, 50 μL of *E. coli* or *S. aureus* bacterial suspension at 1.0 × 10^4^ CFU/mL was added and mixed well. After solidification, 10 mL of additional LB agar medium was added, and three Oxford cups (6 mm in diameter, 10 mm in height) were immediately placed vertically to form an equilateral triangle. Then 100 μL of chemical solution, absolute ethanol, or 50 μg/mL tetracycline solution was added dropwise into the Oxford cups. Absolute ethanol was used as a negative control, and tetracycline was used as a positive control. After the double-layer agar plates were placed in a biochemical incubator at 37 °C for 10–12 h, the diameter of the inhibition zone formed around the Oxford cup was measured. Each test compound was repeated at least 10 times.

### 2.8. Statistical Analysis

All data processing and graph generation were completed with SPSS 21.0 (IBM Inc., Chicago, IL, USA) statistical analysis software and the GraphPad Prism 9.0 (GraphPad Software Inc., La Jolla, CA, USA) program. The data are expressed as the mean ± standard error (SE). The emission dynamics of volatiles released by larvae from different developmental stages and their external immune efficacy were analyzed via one-way analysis of variance (ANOVA) accompanied by the Student–Newman–Keuls (SNK) test for pairwise comparisons. Student’s *t* test was used to compare the emissions of key volatiles between larvae under pathogen stress and those without stress. The level of significance was set at *p* < 0.05 for all the statistical analyses.

## 3. Results

### 3.1. Immune Defensive Efficacy of RPW Larval Volatiles

**Figure 3:**
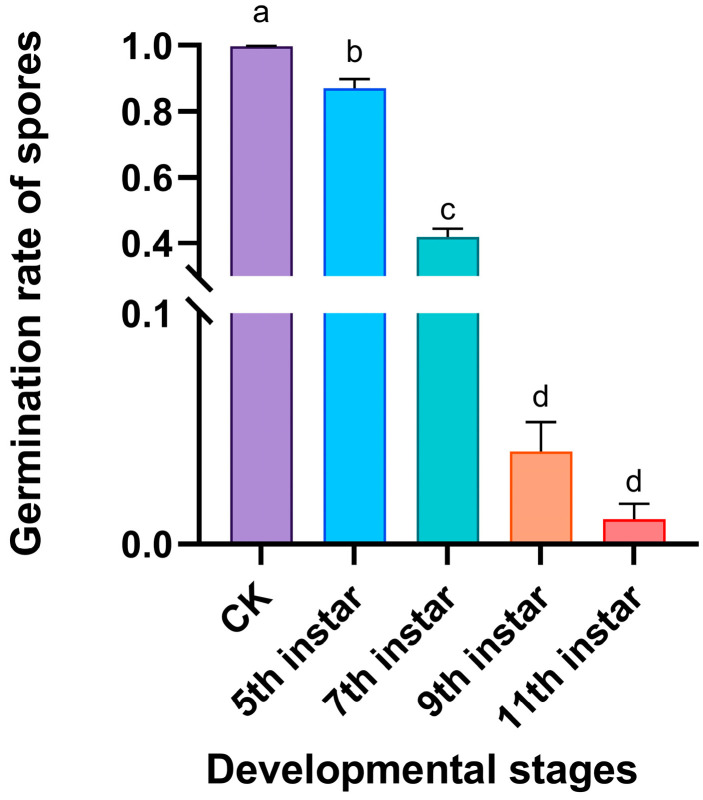
Effects of external volatiles released by RPW larvae from different developmental stages on the germination of *M. anisopliae* spores. The graph shows the mean ± standard error. Bars labeled with different lowercase letters indicate statistically significant differences in the spore germination rate among the different developmental stages (one-way ANOVA followed by Student–Newman–Keuls multiple comparisons at *p* < 0.05).

**Figure 4:**
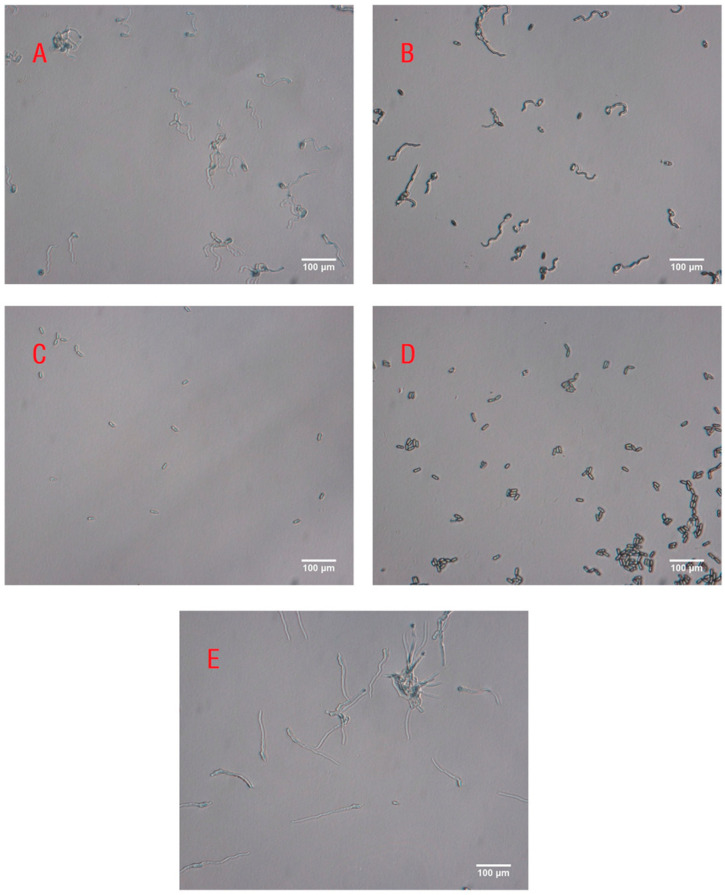
Optical microscopy images of *M. anisopliae* spores exposed to RPW volatiles from (**A**) fifth-instar larvae, (**B**) seventh-instar larvae, (**C**) ninth-instar larvae, and (**D**) eleventh-instar larvae or (**E**) no volatiles as the control group. The scale bar represents 100 μm.

There were significant differences in the inhibitory activity of volatiles released by RPW larvae at different developmental stages against the pathogen *M. anisopliae* (*F*_4,20_ = 617.980, *p* < 0.001; Figure 3). Compared with those in the control group, the volatiles from the fifth-, seventh-, ninth-, and eleventh-instar larvae inhibited the growth of *M. anisopliae* to a certain extent, as the germ tubes of some spores were very short and curved (Figure 4). We further found that with increasing larval development (fifth, seventh, ninth, and eleventh instars), the spore germination rate of *M. anisopliae* significantly decreased to 86.9%, 41.9%, 4.0%, and 1.1%, respectively; however, there was no significant difference in the inhibition on the germination of fungal spores between volatiles from ninth-instar larvae and eleventh-instar larvae (Figure 3). This finding indicated that during larval growth and development, the volatiles of the larvae exhibited increasing immune defensive efficacy, which improved the ability of the individual larvae to respond to pathogen infection.

### 3.2. Volatiles and Emission Dynamics of RPW Larvae Across Developmental Stages

**Figure 5:**
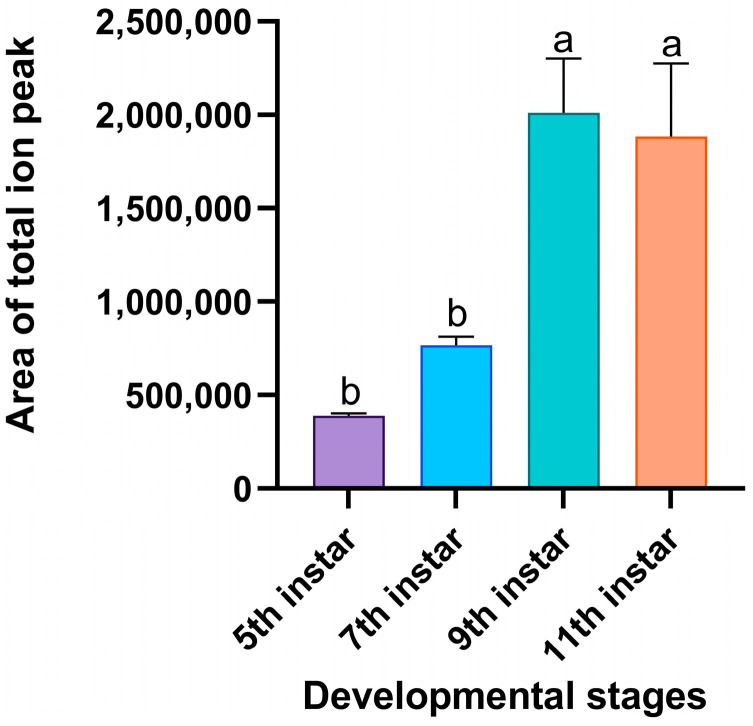
Total peak areas of external volatiles released by RPW larvae of different developmental stages in the chromatograms. The area of the ion peak in the mass spectrometry data calculated via a mathematical integral represents the abundance of volatiles. The graph shows the mean ± standard error. Bars labeled with different lowercase letters indicate statistically significant differences in the total peak area among the different developmental stages (one-way ANOVA followed by Student–Newman–Keuls multiple comparisons at *p* < 0.05).

Significant differences were observed in the total emissions of volatiles released by the RPW larvae at different developmental stages (*F*_3,8_ = 11.004, *p* = 0.003; Figure 4). GC-MS revealed that as the larvae developed, the number of ion peaks and the peak values of individual components both increased (Figure S1). Compared with the external volatiles of fifth-instar larvae, the total ion peak areas of the volatiles from ninth-instar larvae and eleventh-instar larvae were significantly increased by 4.15-fold and 3.83-fold, respectively (Figure 5). These findings indicated that significantly higher abundance levels of volatiles were released by old larvae than by young larvae.

**Figure 6:**
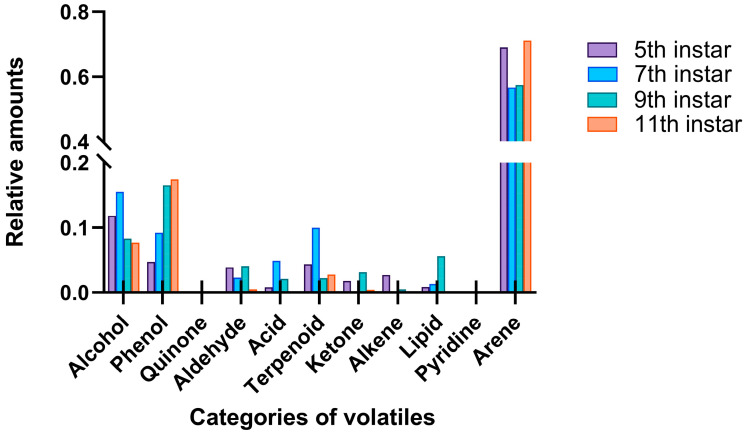
Comparison of the categories and proportions of chemical components enriched with RPW larval volatiles.

A total of 58 chemical components in the volatiles released by larvae at different developmental stages were identified, with 37, 38, 37, and 35 compounds detected in the fifth-, seventh-, ninth-, and eleventh-instar larvae, respectively (Table 1). These volatile compounds were mainly alcohols, phenols, and aromatic hydrocarbons, released by all four larval stages and accounting for 81.39–96.16% of the overall volatile emissions. Other compounds included quinones, aldehydes, acids, terpenes, ketones, alkanes, esters, and pyridines (Figure 6). In particular, the emission of aromatic hydrocarbons was the highest, accounting for 69.02%, 56.69%, 57.50%, and 71.10% of the external volatiles of the fifth-, seventh-, ninth-, and eleventh-instar larvae, respectively (Figure 6).

Qualitative and quantitative analyses of the volatile components showed that the RPW larvae of different developmental stages released 20 common volatile compounds, including *n*-nonanol, 2-phenylethanol, 4-ethylguaiacol, guaiacol, 4-ethylphenol, phenylacetaldehyde, hexanal, benzaldehyde, *n*-decanal, α-pinene, *n*-hexyl acetate, styrene, *p*-xylene, *p*-dichlorobenzene, *o*-xylene, naphthalene, *m*-xylene, ethylbenzene, acetophenone, and butylated hydroxytoluene (Table 1). Among them, styrene was predominant, and the average relative content significantly increased with larval development, with values of 27.09%, 28.04%, 38.60%, and 64.00% in fifth-, seventh-, ninth-, and eleventh-instar larvae, respectively (Table 1). Interestingly, the volatiles of the larvae at different developmental stages also included several unique components. Vanillin, hexanoic acid, geosmin, octanal, and camphorquinone were unique to the external volatiles of the fifth-instar larvae; 1-pentanol, 1-tetradecanol, linalool, and 2-methylnaphthalene were unique to the external volatiles of the seventh-instar larvae; *γ*-octalactone, acetoin, ethyl 2-methylbutyrate, *γ*-decanolactone, 2-*n*-propylpyridine, and benzyl alcohol were unique to the external volatiles of the ninth-instar larvae; and isoeugenol, geraniol, eugenol, 3-amino-4-methylpyridine, and methyl eugenol were unique to the external volatiles of the eleventh-instar larvae (Table 1).

The external volatiles of the RPW larvae thus exhibited different emission patterns and dynamics in response to changes in the developmental stage. The components of these volatiles varied, and their relative contents were quite different.

### 3.3. Effects of Pathogen Stress on Volatile Chemicals in RPW Larvae

**Figure 7:**
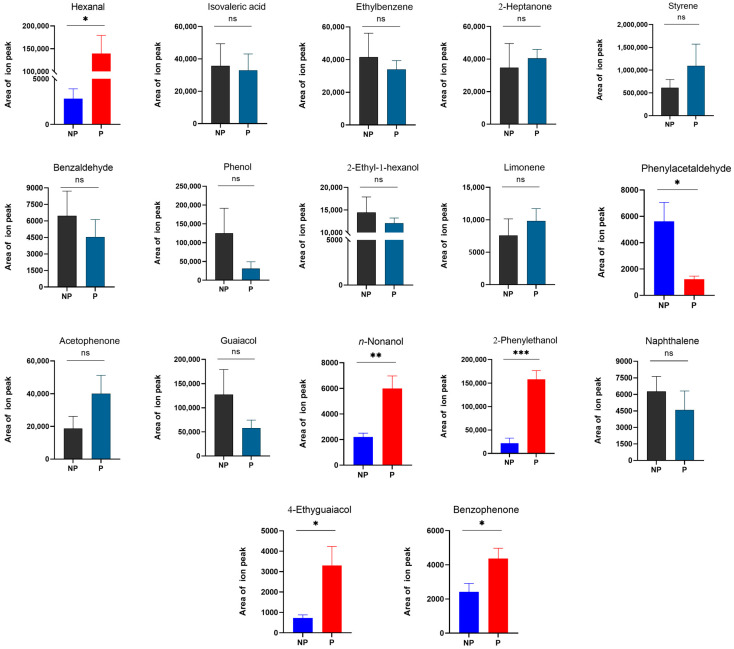
Changes in key volatile emissions from RPW larvae under the environmental stress of pathogens. P, pathogen stress; NP, non-pathogen stress. The graph shows the mean ± standard error. Bars for the same compound labeled with asterisks indicate statistically significant differences in the area of the ion peak under no stress and *M. anisopliae* stress (two-tailed Student’s *t* test; *, *p* < 0.05; **, *p* < 0.01; ***, *p* < 0.001; ns, not significant). The area of the mass spectrometry ion peak was calculated via a mathematical integral and represents the abundance of compounds.

When RPW larvae were infected with *M. anisopliae*, the emission levels of several key volatile compounds changed. It was noted that the average emission of phenylacetaldehyde (*t*_6_ = 3.032, *p* = 0.023) significantly decreased 3.59-fold, whereas those of hexanal (*t*_6_ = −3.420, *p* = 0.014), *n*-nonanol (*t*_6_ = −3.754, *p* = 0.009), 2-phenylethanol (*t*_6_ = −6.390, *p <* 0.001), 4-ethylguaiacol (*t*_6_ = −2.714, *p* = 0.035), and benzophenone (*t*_6_ = −2.489, *p* = 0.047) significantly increased 48.09-, 1.73-, 6.24-, 3.55-, and 0.81-fold, respectively (Figure 7). These compounds were also common volatiles released by larvae at different developmental stages in the absence of stress, and the average relative concentrations were shown in Table 1. For the other volatile compounds [isovaleric acid (*t*_6_ = 0.163, *p* = 0.876), ethylbenzene (*t*_6_ = 0.475, *p* = 0.651), 2-heptanone (*t*_6_ = −0.360, *p* = 0.731), styrene (*t*_6_ = −0.945, *p* = 0.381), benzaldehyde (*t*_6_ = 0.703, *p* = 0.508), phenol (*t*_6_ = 1.374, *p* = 0.219), 2-ethyl-1-hexanol (*t*_6_ = 0.650, *p* = 0.540), limonene (*t*_6_ = −0.696, *p* = 0.512), acetophenone (*t*_6_ = −1.600, *p* = 0.161), guaiacol (*t*_6_ = 1.289, *p* = 0.245), and naphthalene (*t*_6_ = 0.767, *p* = 0.472)], there was no significant difference in abundance (Figure 7).

The upregulated volatile compounds in larvae in response to pathogen stress were the focus of this study, while the downregulated components were not being considered for the time being. According to the above analysis results, the immune function-related candidates *n*-nonanol, 4-ethylguaiacol, 2-phenylethanol, hexanal, and benzophenone were potential active volatile components.

### 3.4. Antimicrobial Functions of Potential Immunocompetent Chemicals

**Figure 8:**
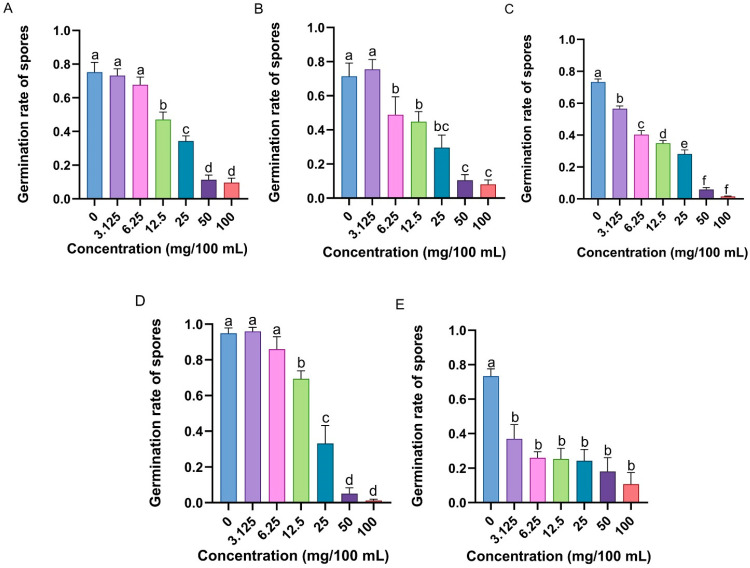
Antifungal activity of potential chemical compounds that exert external immune functions in RPW larval volatiles, including (**A**) *n*-nonanol, (**B**) 4-ethylguaiacol, (**C**) 2-phenylethanol, (**D**) hexanal, and (**E**) benzophenone, measured as the germination rate of *M. anisopliae* spores. The graph shows the mean ± standard error. Bars for the same chemical labeled with different lowercase letters indicate statistically significant differences in the spore germination rate among different concentrations (one-way ANOVA followed by Student–Newman–Keuls multiple comparisons at *p* < 0.05).

The germination of *M. anisopliae* spores was inhibited by specific concentrations of the candidate compounds (Figures S2–S6). The spore germination rate of *M. anisopliae* significantly decreased with increasing *n*-nonanol (*F*_6,35_ = 47.584, *p* < 0.001), 4-ethylguaiacol (*F*_6,35_ = 16.180, *p* < 0.001), 2-phenylethanol (*F*_6,35_ = 193.118, *p* < 0.001), hexanal (*F*_6,35_ = 62.554, *p* < 0.001), and benzophenone (*F*_6,35_ = 10.067, *p* < 0.001) concentrations (Figure 8). Notably, the MICs of these five potentially immunocompetent volatile compounds were 12.5 mg/100 mL (Figure 8A), 6.25 mg/100 mL (Figure 8B), 3.125 mg/100 mL (Figure 8C), 12.5 mg/100 mL (Figure 8D), and 3.125 mg/100 mL (Figure 8E), respectively.

**Figure 9:**
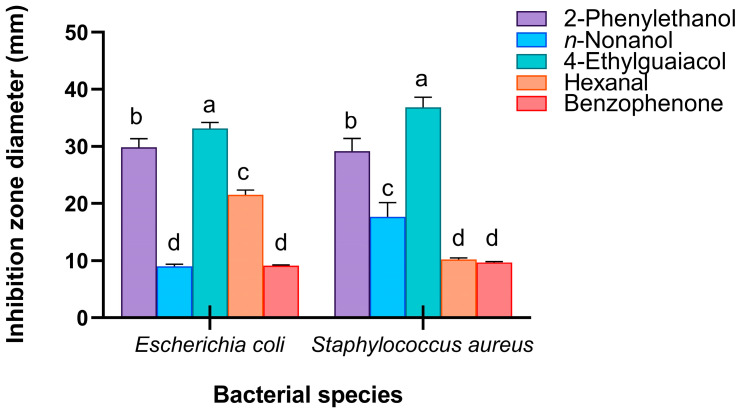
Antibacterial activity of potential chemical compounds that exert external immune functions in RPW larval volatiles, measured as the diameter of the inhibition zone on agar plates. The graph shows the mean ± standard error. Lowercase letters indicate statistically significant differences in the diameter of the inhibition zone among different chemicals (one-way ANOVA followed by Student–Newman–Keuls multiple comparisons at *p* < 0.05).

In addition, these five candidate compounds formed a significant inhibition zone around the Oxford cup (Figures S7 and S8). Surprisingly, the antibacterial activities of 4-ethylguaiacol against *E. coli* (*F*_4,104_ = 187.523, *p <* 0.001) and *S. aureus* (*F*_4,89_ = 106.139, *p <* 0.001) were significantly greater than those of *n*-nonanol, 2-phenylethanol, hexanal, and benzophenone, with average inhibition zone diameters of 33.17 mm and 36.86 mm, respectively (Figure 9).

These results suggest that *n*-nonanol, 4-ethylguaiacol, 2-phenylethanol, hexanal, and benzophenone can inhibit the growth of microorganisms in vitro and are active components and key factors in the volatiles of RPW larvae that exert external immune function, revealing that body volatiles with broad-spectrum immune defensive efficacy play important roles in the process of individual defense against pathogenic infection.

## 4. Discussion

As a common entomopathogen, *M. anisopliae* has been used to prepare a commercial fungal insecticide and is widely used in the biological control of various pests [45]. Similarly, *M. anisopliae* strains isolated from naturally diseased and dead RPWs have been shown to be pathogenic to larvae [17]. However, for the first time, in the present study, volatiles from the RPW larvae were found to inhibit the germination of the *M. anisopliae* spores, which confirmed the external immune function of the insect volatiles. These findings are strikingly similar to those reported by Pu et al. [44] for the oral secretions of the RPW larvae and explain why the food and residues that the larvae have come into contact with are not prone to rot and mold and why the living environment emits a special odor.

In the red flour beetle, *Tribolium castaneum*, the total amount of volatiles produced by adults increases with increasing population density [46]. As expected, the total emission and external inhibitory efficacy of RPW larval volatiles showed a simultaneous increase with instar, and the chemical components and relative amounts also responded to changes in developmental stage and pathogen stress with marked differences. However, whether other factors affect the volatile emission patterns of RPW larvae remains to be further explored.

Some of the volatile compounds found in the RPW larvae are consistent with those present in other insects. Wei et al. [31] reported that the main volatile components in *L. migratoria* were phenylacetonitrile, benzaldehyde, guaiacol, phenol, and 4-vinylanisole. Gross et al. [34] identified compounds including phenol, α-pinene, benzaldehyde, and phenylacetaldehyde among the external volatiles of *H. axyridis*. Obviously, the common components of the external volatiles of RPW larvae at different developmental stages also include guaiacol, α-pinene, benzaldehyde, and phenylacetaldehyde. Notably, styrene was identified as a core volatile shared by all larval developmental stages, and its origin from the larvae rather than lab contamination is supported by two key pieces of evidence. Firstly, styrene was not detectable in the blank control group due to the peak being too small, excluding contamination from lab air, glassware, or sealing film. Secondly, styrene’s relative content showed a consistent developmental trend—gradually increasing from 27.09% (5th instar) to 64.00% (11th instar)—a hallmark of biologically synthesized volatiles, distinct from random contaminant fluctuations. Previous studies have shown that these chemical compounds have inhibitory effects on different types of microorganisms. At a certain concentration, guaiacol can inhibit the growth of *Aspergillus carbonarius* and *Penicillium* [35]; α-pinene acts as an antifungal agent and can inhibit the growth of *Candida albicans* [47]; benzaldehyde can inhibit the growth of *E. coli*, *M. luteus*, and *Saccharomyces* [34]; and phenylacetaldehyde can inhibit the growth of *S. aureus*, *E. coli*, *Salmonella typhimurium*, *Streptococcus pneumoniae*, *Erysipelothrix rhusiopathiae*, and *Saccharomyces cerevisiae* [48]. Accordingly, it is speculated that the body volatiles present in the RPW larvae can also defend against infection by other specific pathogens in addition to *M. anisopliae*, which may play a more extensive role in external immune defense.

Owing to the individual stress response, the expression levels of some components involved in the volatile emissions of insects under pathogen stress transiently change, and these components may perform specific immune functions. The results of this study revealed that after RPW larvae were infected with a sublethal dose of *M. anisopliae*, the abundances of five volatile compounds, namely, *n*-nonanol, 4-ethylguaiacol, 2-phenylethanol, hexanal and benzophenone, were significantly increased. 2-Phenylethanol not only has certain inhibitory efficacy against various postharvest fruit fungal diseases such as *Penicillium italicum* but also can have some inhibitory efficacy against *Colletotrichum camelliae* and *Chaetomium globosum* [49,50]. As a good antifungal agent, hexanal has an inhibitory effect on *Aspergillus niger*, and its fumigant can also effectively reduce the spore viability of *Penicillium* [51,52]. Benzophenone has antifungal activity against *C. albicans* and *A. niger* [53]. Similarly, the bioactivity of *n*-nonanol, 4-ethylguaiacol, 2-phenylethanol, hexanal, and benzophenone on the germination of *M. anisopliae* spores were investigated in this study, and the results clarified their potential efficacy in host defense against pathogenic fungal infection. Surprisingly, these five key volatile compounds also significantly inhibited the growth of bacteria at a certain concentration. Therefore, the volatiles of RPW larvae have efficient and broad-spectrum immune defense properties.

Interestingly, insect volatiles function primordially as pheromones [31,32], whereas our study revealed that they have another effect, that is, immune function. This new finding in RPW is different from that of Yin et al. [39] on banana corm weevils *Cosmopolites sordidus*, which belong to the same family (Curculionidae). This immune function helps larvae reduce infection risk by cleaning their feeding tunnels with volatiles. Notably, adults use similar volatiles as aggregation pheromones [54]. Thus, these compounds serve dual critical roles across life stages—both coordinating reproduction and maintaining microenvironmental health.

## 5. Conclusions

In summary, the present study contributes important information for exploring the emission dynamics of the chemical components of volatiles from RPW larvae during development and changes in pathogen stress. Furthermore, this study provides important chemical cues for understanding the mechanisms of immune system coding in RPW larvae. In this study, we clearly revealed potential volatile immunoactive compounds, including *n*-nonanol, 4-ethylguaiacol, 2-phenylethanol, hexanal, and benzophenone. Related discoveries will be useful in developing environmentally friendly immune regulation technology for RPW management.

## Figures and Tables

**Table 1:** Chemical components and emission patterns of external volatiles collected from the different RPW larval developmental stages.

No.	Chemical Compounds ^a^	Molecular Formula	Average Relative Amounts (%)			
			5th-Instar	7th-Instar	9th-Instar	11th-Instar
1	Verbenol	C_10_H_16_O	ND ^b^	0.16	0.02	ND
2	*n*-Nonanol	C_9_H_20_O	1.63	0.30	0.27	0.35
3	Linalool	C_10_H_18_O	ND	0.25	ND	ND
4	Geraniol	C_10_H_18_O	ND	ND	ND	0.33
5	Geosmin	C_14_H_7_OF_3_Cl_2_	0.22	ND	ND	ND
6	Benzyl alcohol	C_7_H_8_O	ND	ND	0.01	ND
7	*α*-Terpineol	C_10_H_18_O	ND	0.40	ND	0.03
8	2-Phenylethanol	C_5_H_12_O_2_	7.55	5.08	7.26	6.82
9	2-Ethyl-1-hexanol	C_10_H_16_O_2_	1.60	1.39	0.75	ND
10	1-Tetradecanol	C_14_H_30_O	ND	0.77	ND	ND
11	1-Pentanol	C_5_H_12_O	ND	4.07	ND	ND
12	1-Dodecanol	C_50_H_57_N_5_O_13_S_5_	0.81	0.12	ND	0.03
13	1-Undecanol	C_11_H_24_O	ND	2.93	ND	0.10
14	Phenol	C_6_H_6_O	0.78	0.07	8.22	ND
15	4-Ethylguaiacol	C_9_H_12_O_2_	0.39	1.17	0.19	3.92
16	Methyl eugenol	C_11_H_14_O_2_	ND	ND	ND	0.08
17	Isoeugenol	C_10_H_12_O_2_	ND	ND	ND	0.47
18	Guaiacol	C_7_H_8_O_2_	3.04	7.85	7.82	2.66
19	4-Ethylphenol	C_20_H_12_Br_2_O_2_	0.39	0.13	0.21	5.88
20	2,3-Xylenol	C_11_H_10_N_4_Cl_2_	0.12	ND	0.07	4.29
21	Eugenol	C_10_H_12_O_2_	ND	ND	ND	0.11
22	Camphorquinone	C_10_H_14_O_2_	0.08	ND	ND	ND
23	Vanillin	C_8_H_8_O_3_	1.38	ND	ND	ND
24	Phenylacetaldehyde	C_8_H_8_O	0.12	0.94	0.12	0.05
25	Octanal	C_8_H_16_O	0.17	ND	ND	ND
26	Hexanal	C_6_H_12_O	1.01	0.23	3.63	0.19
27	Benzaldehyde	C_7_H_6_O	0.81	0.99	0.27	0.22
28	*n*-Decanal	C_10_H_20_O	0.40	0.13	0.02	0.01
29	Isovaleric acid	C_5_H_10_O_2_	0.57	4.89	2.12	ND
30	Hexanoic acid	C_6_H_12_O_2_	0.24	ND	ND	ND
31	*β*-Pinene	C_10_H_16_	ND	ND	1.03	1.38
32	*α-*Pinene	C_10_H_16_	4.33	6.73	1.16	1.35
33	Borneol	C_10_H_18_O	ND	2.49	0.04	ND
34	2-Methylisoborneol	C_11_H_20_O	ND	0.73	ND	0.04
35	Benzophenone	C_13_H_10_O	0.59	ND	0.13	0.26
36	Acetoin	C_4_H_8_O_2_	ND	ND	0.78	ND
37	2-Heptanone	C_7_H_14_O	1.21	ND	2.21	0.17
38	Limonene	C_10_H_16_	2.68	0.18	0.49	ND
39	*n*-Hexyl acetate	C_8_H_16_O_2_	0.63	0.85	0.12	0.09
40	Methyl salicylate	C_8_H_8_O_3_	0.23	0.46	ND	ND
41	*γ*-Octalactone	C_7_H_7_N_3_	ND	ND	4.90	ND
42	*γ*-Decanolactone	C_10_H_18_O_2_	ND	ND	0.16	ND
43	Ethyl-2-methylbutyrate	C_7_H_14_O_2_	ND	ND	0.43	ND
44	3-Amino-4-methylpyridine	C_6_H_8_N_2_	ND	ND	ND	0.09
45	2-*n*-Propylpyridine	C_8_H_11_N	ND	ND	0.04	ND
46	Toluene	C_7_H_8_	5.04	0.61	4.58	ND
47	Styrene	C_8_H_8_	27.09	28.04	38.60	64.00
48	*p*-Xylene	C_8_H_10_	8.32	8.21	4.76	0.45
49	*p*-Dichlorobenzene	C_6_H_4_Cl_2_	0.04	0.18	0.04	0.08
50	*o*-Xylene	C_8_H_10_	3.00	2.44	1.84	0.52
51	Naphthalene	C_10_H_8_	3.75	4.54	0.30	0.47
52	*m*-Xylene	C_8_H_10_	7.36	6.98	4.22	0.36
53	Indole	C_9_H_8_NBr	8.06	0.29	ND	1.25
54	Ethylbenzene	C_10_H_15_NO_2_	2.88	2.33	2.50	0.45
55	Acetophenone	C_8_H_8_O	2.13	1.92	0.60	3.45
56	1,2,4,5-Tetramethylbenzene	C_10_H_14_	0.21	0.32	ND	0.06
57	Butylated hydroxytoluene	C_15_H_24_O	1.14	0.63	0.05	0.01
58	2-Methylnaphthalene	C_11_H_10_	ND	0.19	ND	ND

^a^ Compounds were identified by comparison of their retention time and mass spectrometry spectrum with those of authentic compounds in the FFNSC 1.2 library. ^b^ ND: not detectable (absent or peak too small to determine composition).

## Data Availability

The data are available in a publicly accessible repository. The original data presented in this study are openly available in FigShare at https://doi.org/10.6084/m9.figshare.30664613.

## Supplementary Materials

The following supporting information can be downloaded at: https://www.mdpi.com/article/10.3390/insects16121266/s1. Figure S1: Total ion chromatograms (TICs) of red palm weevil volatiles from (A) fifth-instar larvae, (B) seventh-instar larvae, (C) ninth-instar larvae, and (D) eleventh-instar larvae separated via gas chromatography-mass spectrometry. Figure S2: Optical microscopy images of *Metarhizium anisopliae* spores exposed to *n*-nonanol at concentrations of (A) 1 mg/mL, (B) 0.5 mg/mL, (C) 0.25 mg/mL, (D) 0.125 mg/mL, (E) 0.0625 mg/mL, and (F) 0.03125 mg/mL, with (G) 0 mg/mL as the control. The scale bar represents 100 μm. Figure S3: Optical microscopy images of *Metarhizium anisopliae* spores exposed to 4-ethylguaiacol at concentrations of (A) 1 mg/mL, (B) 0.5 mg/mL, (C) 0.25 mg/mL, (D) 0.125 mg/mL, (E) 0.0625 mg/mL, and (F) 0.03125 mg/mL, with (G) 0 mg/mL as the control. The scale bar represents 100 μm. Figure S4: Optical microscopy images of *Metarhizium anisopliae* spores exposed to 2-phenylethanol at concentrations of (A) 1 mg/mL, (B) 0.5 mg/mL, (C) 0.25 mg/mL, (D) 0.125 mg/mL, (E) 0.0625 mg/mL, and (F) 0.03125 mg/mL, with (G) 0 mg/mL as the control. The scale bar represents 100 μm. Figure S5: Optical microscopy images of *Metarhizium anisopliae* spores exposed to hexanal at concentrations of (A) 1 mg/mL, (B) 0.5 mg/mL, (C) 0.25 mg/mL, (D) 0.125 mg/mL, (E) 0.0625 mg/mL, and (F) 0.03125 mg/mL, with (G) 0 mg/mL as the control. The scale bar represents 100 μm. Figure S6: Optical microscopy images of *Metarhizium anisopliae* spores exposed to benzophenone at concentrations of (A) 1 mg/mL, (B) 0.5 mg/mL, (C) 0.25 mg/mL, (D) 0.125 mg/mL, (E) 0.0625 mg/mL, and (F) 0.03125 mg/mL, with (G) 0 mg/mL as the control. The scale bar represents 100 μm. Figure S7: Representative zones of inhibition against *Escherichia coli* formed by (A) *n*-nonanol, (B) 4-ethylguaiacol, (C) 2-phenylethanol, (D) hexanal, and (E) benzophenone. Absolute ethanol and tetracycline were used as negative and positive controls, respectively, for each plate. Figure S8: Representative zones of inhibition against *Staphylococcus aureus* formed by (A) *n*-nonanol, (B) 4-ethylguaiacol, (C) 2-phenylethanol, (D) hexanal, and (E) benzophenone. Absolute ethanol and tetracycline were used as negative and positive controls, respectively, for each plate.
