# Supplementary material for: Chemical Components, Emission Dynamics, and External Immune Functions of Red Palm Weevil Larval Volatiles in Response to Changes in Developmental Stages and Pathogen Stress
Source: Insects. 2025 Dec 13;16(12):1266. doi: 10.3390/insects16121266 (PMC12734331; doi:10.3390/insects16121266)
Supplement: Supplementary file 1 [file insects-16-01266-s001.zip › insects-4035671-supplementary.pdf]

Supplementary materials

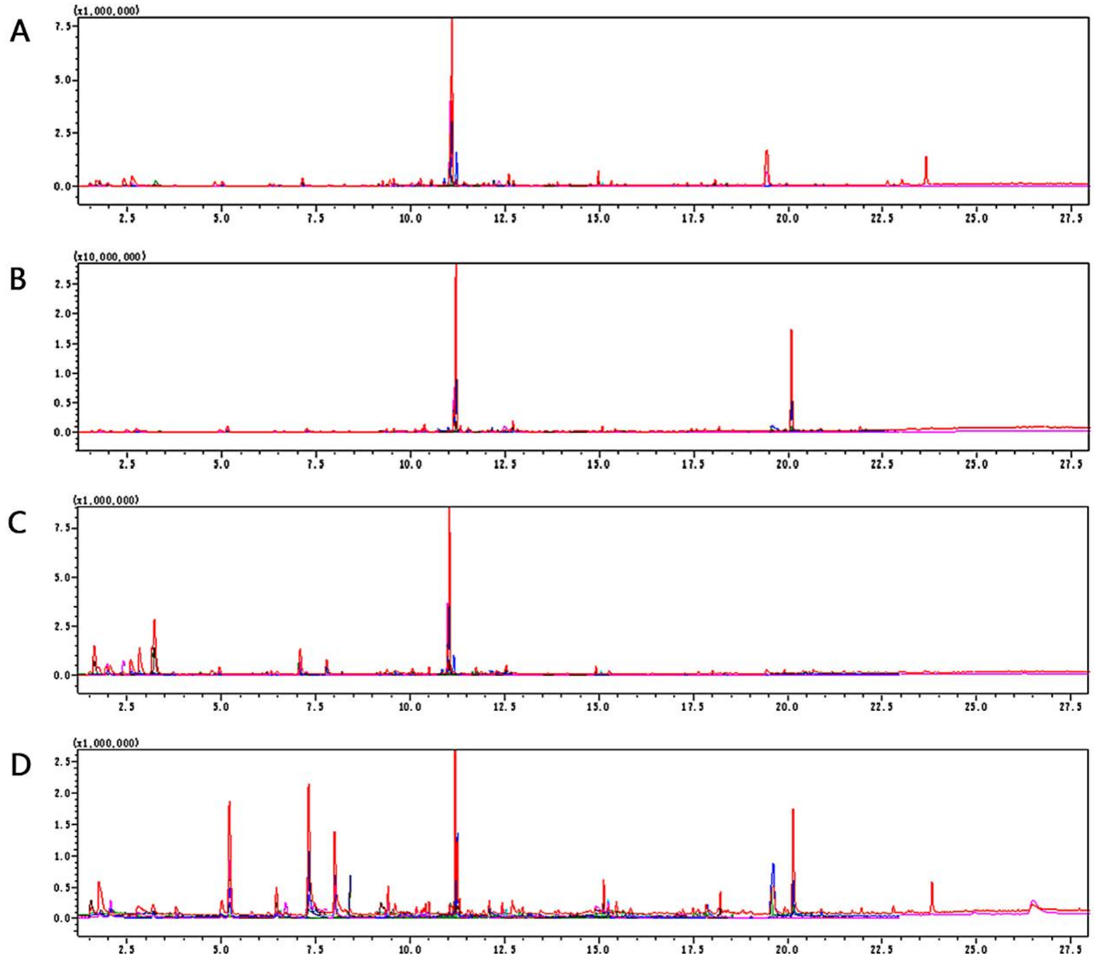

Figure S1 Total ion chromatograms (TICs) of red palm weevil volatiles from (A) fifth-instar larvae, (B) seventh-instar larvae, (C) ninth-instar larvae, and (D) eleventh-instar larvae separated via gas chromatography-mass spectrometry.

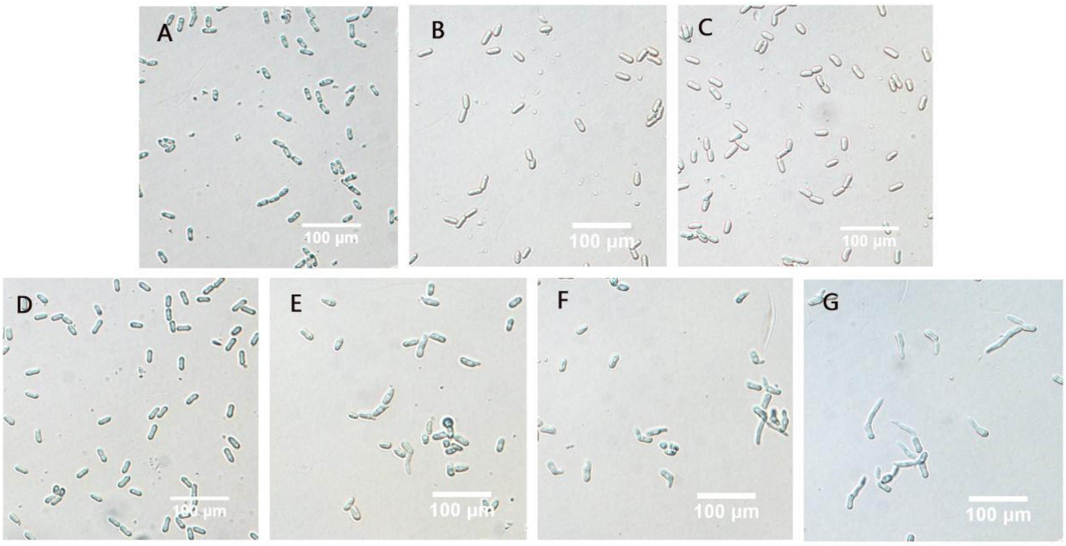

Figure S2 Optical microscopy images of *Metarhizium anisopliae* spores exposed to *n*-nonanol at concentrations of (A) 1 mg/mL, (B) 0.5 mg/mL, (C) 0.25 mg/mL, (D) 0.125 mg/mL, (E) 0.0625 mg/mL, and (F) 0.03125 mg/mL, with (G) 0 mg/mL as the control. The scale bar represents 100 μm.

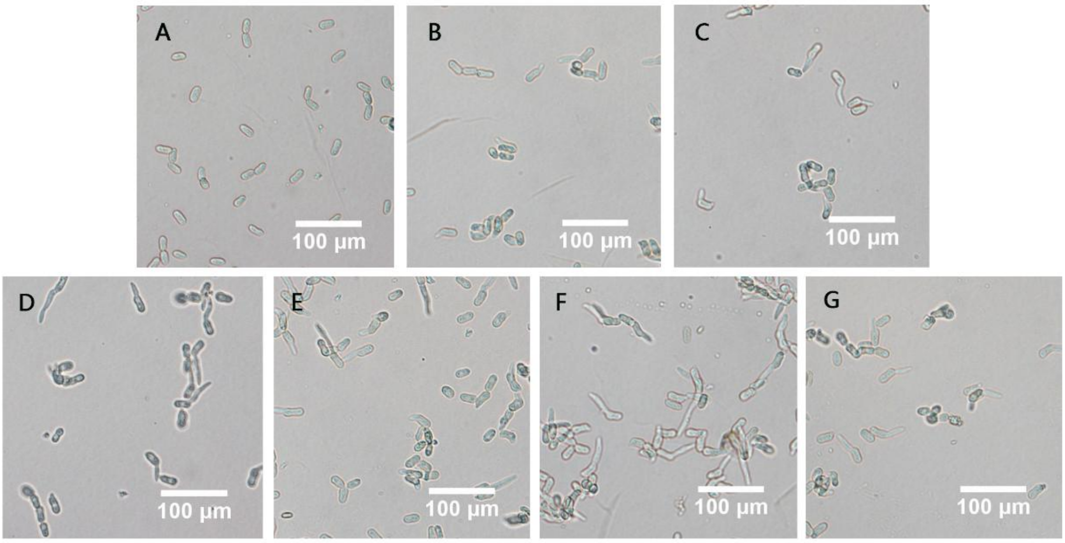

Figure S3 Optical microscopy images of *Metarhizium anisopliae* spores exposed to 4-ethylguaiacol at concentrations of (A) 1 mg/mL, (B) 0.5 mg/mL, (C) 0.25 mg/mL, (D) 0.125 mg/mL, (E) 0.0625 mg/mL, and (F) 0.03125 mg/mL, with (G) 0 mg/mL as the control. The scale bar represents 100 µm.

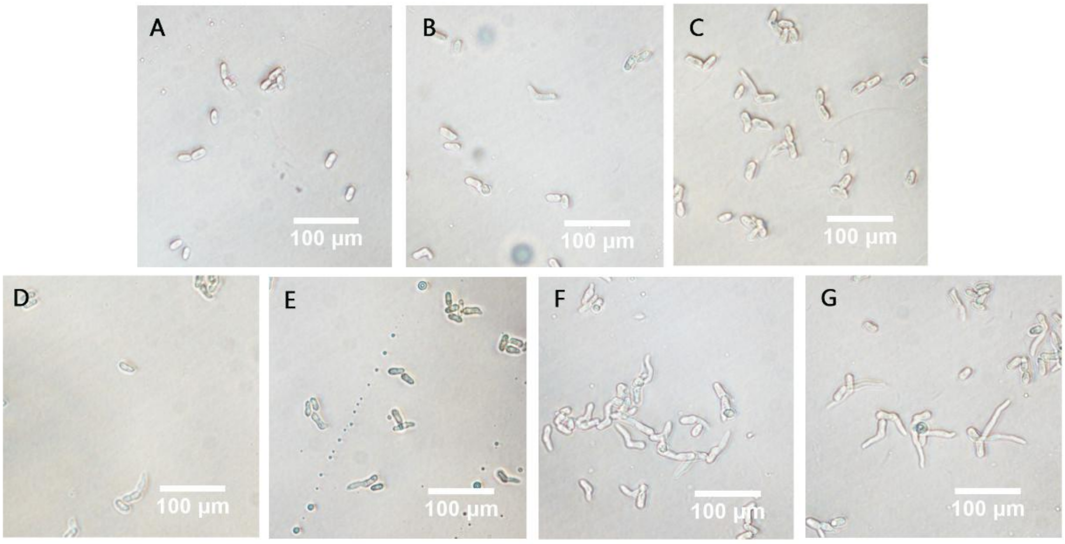

Figure S4 Optical microscopy images of *Metarhizium anisopliae* spores exposed to 2-phenylethanol at concentrations of (A) 1 mg/mL, (B) 0.5 mg/mL, (C) 0.25 mg/mL, (D) 0.125 mg/mL, (E) 0.0625 mg/mL, and (F) 0.03125 mg/mL, with (G) 0 mg/mL as the control. The scale bar represents 100 μm.

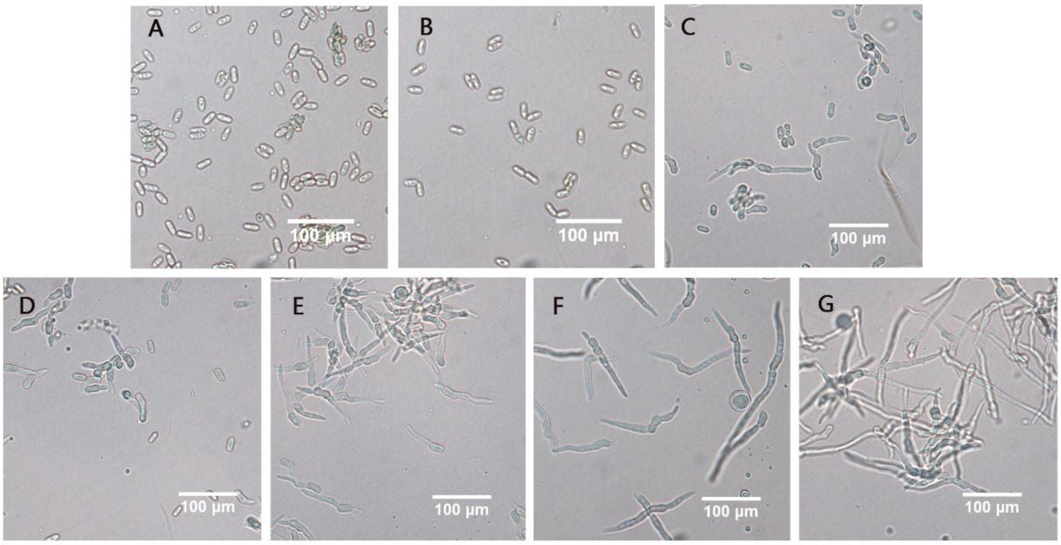

Figure S5 Optical microscopy images of *Metarhizium anisopliae* spores exposed to hexanal at concentrations of (A) 1 mg/mL, (B) 0.5 mg/mL, (C) 0.25 mg/mL, (D) 0.125 mg/mL, (E) 0.0625 mg/mL, and (F) 0.03125 mg/mL, with (G) 0 mg/mL as the control. The scale bar represents 100 µm.

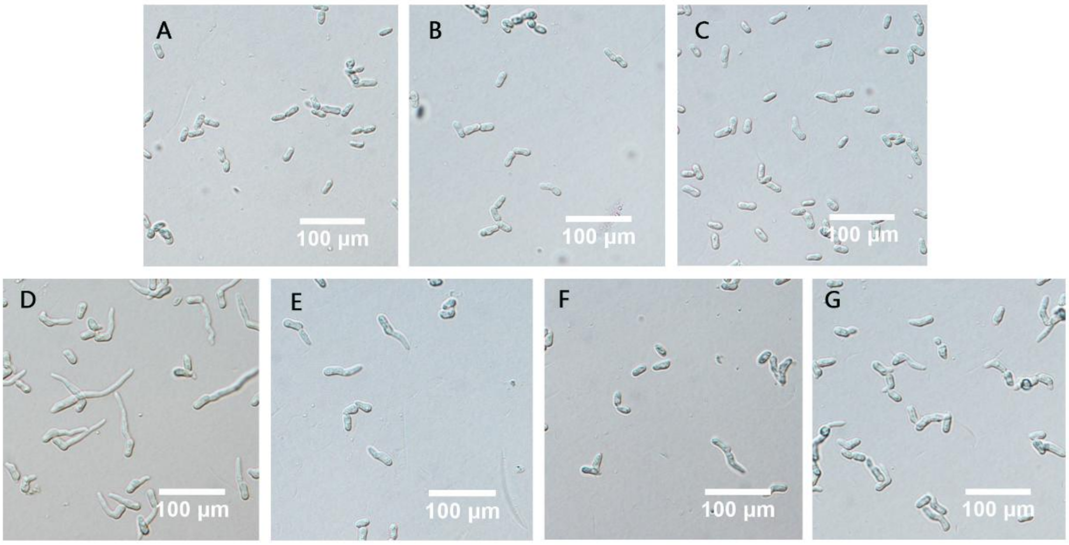

Figure S6 Optical microscopy images of *Metarhizium anisopliae* spores exposed to benzophenone at concentrations of (A) 1 mg/mL, (B) 0.5 mg/mL, (C) 0.25 mg/mL, (D) 0.125 mg/mL, (E) 0.0625 mg/mL, and (F) 0.03125 mg/mL, with (G) 0 mg/mL as the control. The scale bar represents 100 μm.

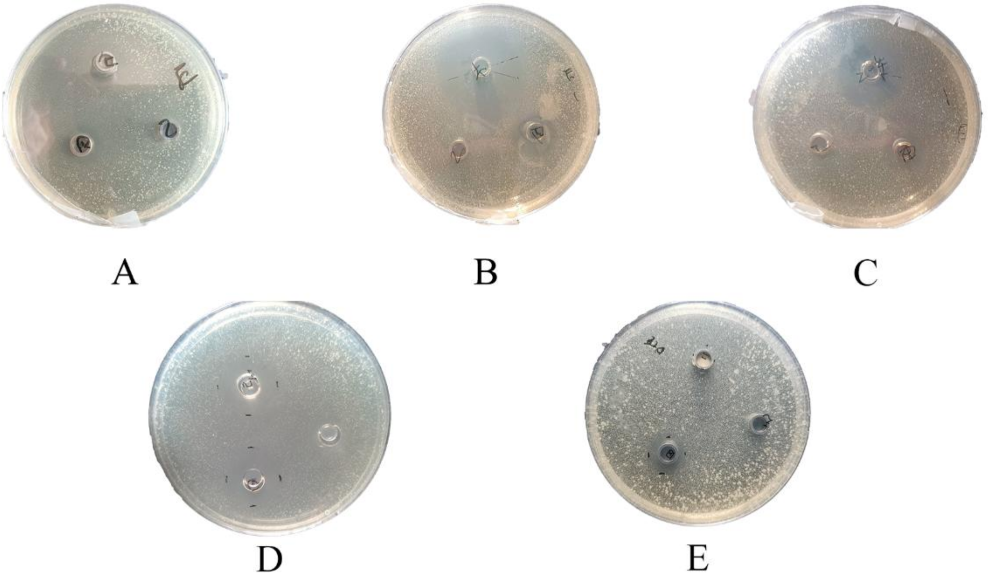

Figure S7 Representative zones of inhibition against *Escherichia coli* formed by (A) *n*-nonanol, (B) 4-ethylguaiacol, (C) 2-phenylethanol, (D) hexanal, and (E) benzophenone. Absolute ethanol and tetracycline were used as negative and positive controls, respectively, for each plate.

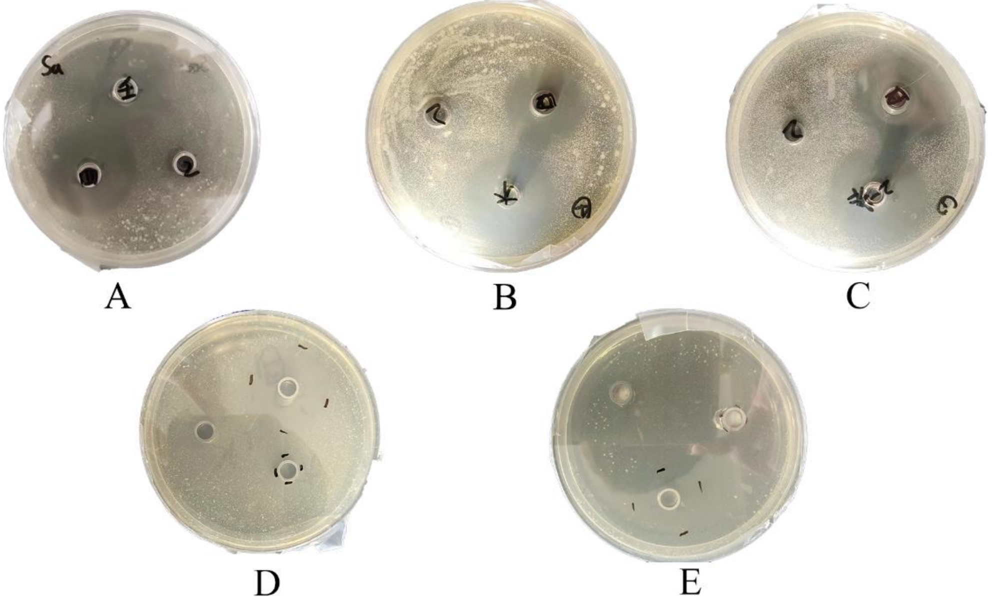

Figure S8 Representative zones of inhibition against *Staphylococcus aureus* formed by (A) *n*-nonanol, (B) 4-ethylguaiacol, (C) 2-phenylethanol, (D) hexanal, and (E) benzophenone. Absolute ethanol and tetracycline were used as negative and positive controls, respectively, for each plate.
